# Image quality of arterial phase and parenchymal blood volume (PBV) maps derived from C-arm computed tomography in the evaluation of transarterial chemoembolization

**DOI:** 10.1186/s40644-018-0151-y

**Published:** 2018-05-02

**Authors:** Tanja Zitzelsberger, Roland Syha, Gerd Grözinger, Sasan Partovi, Konstantin Nikolaou, Ulrich Grosse

**Affiliations:** 1 Department of Diagnostic and Interventional Radiology, University of Tuebingen, Hoppe-Seyler-Straße 3, 72076 Tuebingen, Germany; 2 Department of Radiology, Section of Interventional Radiology, University Hospitals Cleveland Medical Center, Case Western Reserve University, Cleveland, OH USA

**Keywords:** Hepatocellular carcinoma, Transarterial chemoembolization, C-arm computed tomography, Parenchymal blood volume, Image quality

## Abstract

**Background:**

To evaluate the benefits of arterial phase imaging and parenchymal blood volume (PBV) maps acquired by C-arm computed tomography during TACE procedure in comparison to cross-sectional imaging (CSI) using CT or MRI.

**Methods:**

From January 2014 to December 2016, a total of 29 patients with HCC stage A or B (mean age 65 years; range 47 to 81 years, 86% male) were included in this study. These patients were referred to our department for TACE treatment and received peri-interventional C-arm CT. Dual phase findings of each lesion in terms of overall image quality, conspicuity, tumor size and feeding arteries were compared between arterial phase imaging and PBV using 5-point semi-quantitative Likert-scale, whereby pre-interventional CSI served as reference standard.

**Results:**

A significantly higher overall image quality of the PBV maps compared to arterial phase C-arm CT acquisitions (4.34 (±0.55) vs. 3.93 (±0.59), *p* = 0.0032) as well as a higher conspicuity of HCC lesions (4.27 ± 0.74 vs. 3.83 ± 1.08, *p* < 0.0001) was observed. Arterial phase imaging led to an overestimation of tumor size (mean size, 26.5 ± 15.9 mm) compared to PBV (24.9 ± 15.2 mm, *p* = 0.0004) as well as CSI (25.2 ± 15.1 mm), *p* = 0.021). Regarding detectability of tumor feeding arterial vessels, significantly more feeding vessels were detected in arterial phase C-arm CT (*n* = 1.67 ± 0.92 vessels) compared to PBV maps (*n* = 1.27 ± 0.63 vessels) (*p* = 0.0001). One lesion was missed in pre-interventional CT imaging, but detected by C-arm CT.

**Conclusion:**

The combination of PBV maps and arterial phase images acquired by C-arm CT during TACE procedure enables precise detection of the majority of HCC lesions and tumor feeding arteries and has therefore the potential to improve patient outcome.

## Background

Hepatocellular Carcinoma (HCC) is one of the leading cancer entities in the industrialized countries with rising incidence [1]. Within the standard of care, transarterial chemoembolization (TACE) is recommended as first-line therapy in certain patients with compensated liver function in intermediate stage HCC (stage B in the Barcelona Clinic Liver Cancer (BCLC) staging system). TACE may contribute to downstaging or bridging to orthotopic liver transplantation in early stage HCC (BCLC stage A) according to the Barcelona Clinic Liver Cancer (BCLC) staging system [2] and the EASL-EORTC guidelines [3]. The goal of TACE treatment is to embolize the tumor’s arterial blood supply and to deliver high chemotherapeutic drug concentrations to the viable tumor tissue, with the goal to achieve a high rate of tumor necrosis while preserving the surrounding heathy liver parenchyma and diminish systemic toxicity [4]. Therefore, during TACE treatment, several digital subtraction angiography series (DSA) are typically necessary to precisely identify the tumor and its feeding vasculature. In patients with complex hepatic arterial branching patterns, an increased number of DSA acquisitions at different angles are usually required to identify the tumor feeding arteries, which in turn leads to an increased radiation and contrast medium exposure.

In this context, cone beam computed tomography (CBCT) is an evolving and attractive tool, due to its capability of peri-interventional 3D imaging in combination with high vascular contrast and high spatial resolution [5]. In recent studies, CBCT has demonstrated a high diagnostic accuracy for detecting hepatic tumor lesions and small feeding arteries to hepatic neoplasms for the guidance of trans-arterial chemoembolization (TACE) [6–8]. Previous studies have also shown that peri-interventional CBCT during a TACE procedure influences diagnosis as well as treatment in up to 81%, due to improved tumor feeder detection, catheter navigation and treatment effect assessment [9, 10]. An advanced development of these techniques is represented by dual-phase cone-beam computed tomography (CBCTHA), which allows an assessment of post-processed maximum intensity projections and parenchymal blood volume (PBV) maps, in addition to acquiring native and contrast-enhanced images [11]. Promising experiences with these techniques in the initial evaluation and treatment of HCC have been published lately [12–14]. However, it remains unclear which of the images acquired with C-arm CT are suited best to delineate tumor size and tumor feeding arteries.

Therefore, the aim of this retrospective study was to compare the image quality of arterial phase and PBV maps acquired with CBCT during TACE treatment, whereby pre-interventional acquired cross-sectional images (CT or MRI) served as reference standard.

## Methods

### Patients

From January 2014 to December 2016 a total of 29 patients with HCC (mean age at examination: 65 years (range 47 to 81 years), 86% male), which were referred to our department for TACE treatment, received peri-interventional C-arm CT and were included in this retrospective study. Patients with early stage HCC (BCLC A) as well as intermediate stage HCC (BCLC B) were included. Exclusion criteria of this study were in accordance with the CIRSE guidelines [15]:

• Decompensated cirrhosis (Child C).

• Extrahepatic spread.

• Severely reduced portal vein flow.

• Renal insufficiency (creatinine ≥2 mg/dl or creatinine clearance ≤30 ml/min).

• Bilirubin level > 2 mg/dl.

• Advanced hepatic encephalopathy.

Underlying cause for liver cirrhosis and HCC development were Hepatitis-C-virus infection (*n* = 11), alcohol abuse (*n* = 9), NASH (*n* = 2) and hemochromatosis (*n* = 1). In six patients, the underlying cause for developing HCC was cryptogenic. Seventeen patients of this collective had received previous TACE treatment. Detailed patient information is listed in Table 1. The study was approved by the institutional review committee and was in compliance with HIPAA regulations. Due to the retrospective nature of the study, informed consent for retrospective data analysis was waved by the institutional review board.

**Table 1:** Baseline characteristics 1

Characteristics	No. of Patients
Sex	
Male	25
Female	4
Age at examination (median)	
< 65	15
≥ 65	14
Number of tumors	
1	11
2	6
> = 3	6
Tumor size	
< 3 cm	38
3-5 cm	9
> 5 cm	5
BCLC^a^	
A	16
B	13
Cause of liver cirrhosis	
Hepatitis C	11
Alcohol related	9
Hemochromatosis	1
Non-alcoholic steatohepatitis	2
Cryptogenic	6
MELD^b^ score	8.2
Previous TACE^c^	17

^a^Barcelona clinic liver cancer

^b^Model of End Stage Liver Disease

^c^transarterial chemoembolization

### Pre-interventional imaging

All patients underwent cross-sectional imaging (multiphase CT, *n* = 25 or MRI, *n* = 4) before TACE following the national guidelines for the assessment of HCC [16]. Median interval between baseline imaging and TACE was 8 days with a range of 4–22 days. Multiphase CT included a non-enhanced, arterial phase (30s after injection of contrast media) as well as portal venous phase (70s after injection of contrast media). The CT exam was performed on a 128 row detector CT with one or two x-ray tubes (SOMATOM Definition AS+ or Definition Flash, Siemens Healthcare, Forchheim, Germany). MRI consisted of a T2 weighted turbo-spin-echo sequence, an unenhanced and dynamic contrast enhanced T1 weighted gradient-echo sequences (VIBE) as well as diffusion-weighted sequences acquired at a field strength of 1.5 T (Magnetom Avanto fit/ Magnetom Aera, Siemens Germany).

All HCC lesions were classified based on morphology in diffuse (*n* = 9, 17%) or encapsulated (*n* = 43, 83%) lesions. According to LIRADS criteria, encapsulated HCC was defined as a predominantly round lesion with the presence of a capsule and clear wash-out in cross-sectional imaging. Diffuse HCC lesions were defined as predominantly irregular or lobular lesions without a capsule [17]. The number and extent of HCC lesion was noted.

### Transarterial chemoembolization (TACE)

In all patients, endovascular intervention was performed using the same robotic digital subtraction angiography system (Artis Zeego Q, VE 40 A, Siemens, Forchheim, Germany). Percutaneous arterial access was achieved through the common femoral artery (19 G needle) under local anesthesia with placement of a 4F sheath (Terumo, Leuven, Belgium). A 4F straight catheter (Terumo, Leuven, Belgium) was utilized for aortography, while a 4F Cobra (C2) or sidewinder (SIM1) catheter was used for entering the coeliac trunk. A 2.7F coaxial microcatheter (Progreat; Terumo, Leuven, Belgium) was used for selective and super-selective access of the hepatic arteries. In case of extrahepatic tumor supply (two patients with a right inferior phrenic artery and one patient with a lumbar artery supply), an embolization of these additional feeders using pushable microcoils was performed. In all cases, a superselective TACE with DEB (100-300 μm DC-Beads (BTG, Langweid/Augsburg, Germany) loaded with 50 mg Epirubicin was conducted.

### C-arm computed tomography

C-Arm CT consisted of an unenhanced rotation (mask run) and contrast enhanced rotations (return and fill run) with contrast medium injection from the proper hepatic artery for acquisition of parenchymal blood volume (PBV) maps. The following C-Arm CT image acquisition parameters were used: time per rotation 4 s, total examination time 16 s, 200° total angle, per frame 0.8°, 248 frames, matrix 616 × 480, pixel size 616 μm, projection on 30x40cm flat panel. The actual tube current and tube voltage were automatically adjusted to the individual patient by the system. For contrast enhancement, 30 ml diluted contrast medium (7.5 ml Ultravist 370, Bayer Schering, Leverkusen, Germany and 22.5 saline solution) was administered by an automated power injector (Accutron-HP-D, Medtron, Saarbrücken, Germany), using a flow rate of 3 ml/s. Contrast injection was performed immediately after the mask run. Contrast enhanced acquisition was performed in a steady state of liver perfusion [13, 14]. Image reconstruction was conducted on a multimodality workstation (MMWP VD 10, Siemens Healthcare, Forchheim, Germany). Fill run and mask run were subtracted. A non-rigid registration algorithm was performed to mitigate the motion between the two runs. The arterial input function value is calculated from an automated histogram analysis of the vessel tree. This arterial input function value is then applied as a scaling factor to obtain the PBV map [12].

All arterial phases and processed PBV maps were analyzed concerning number and extent of HCC lesions as well as the number of tumor feeding vessels.

### Image analysis

A retrospective analysis of image quality and diagnostic value of arterial phase and PBV maps was performed by two experienced board certified readers (4 and 6 years of experience in dedicated interventional radiology and angiography). For this purpose, standardized reconstructions in the axial plane were acquired and clinical data of the patients were recorded.

The overall image quality of arterial phase C-arm CT and PBV maps as well as the conspicuity of HCC lesion was evaluated on a 5-point semi-quantitative Likert-scale: (1) non diagnostic image quality; (2) poor image quality; (3) moderate image quality; (4) good image quality; (5) excellent image quality.

Moreover, a 5-point Likert-Scale was used to assess the presence of artifacts (1 = compromising diagnostic image quality, 2 = present, but not compromising diagnostic image quality, 3 = no artifacts presents) and noise (1 = severe, 2 = moderate, 3 = mild, 4 = minimal, 5 = none). In addition, the delineation of central hepatic arteries as well as the visualization of the gallbladder wall was evaluated: (1) marked blurring of organ contours, (2) subtle blurring of organ contours, (3) moderate delineation of organ contours, (4) very good delineation of organ contours and (5) excellent delineation of organ contours. Complete covering of the entire liver on C-arm CT was assessed by: (1) non diagnostic, (2) incomplete but diagnostically irrelevant, (3) entire liver covered, diagnostic standard.

Tumor size was determined based on maximum diameter on axial images and was measured in arterial phase, PBV map and cross sectional images (CSI). As a quantitative measure, the contrast-to-noise ratio (CNR) was computed using the following formula (μ_R_ and μ_L_ represent the mean values of the ROI and normal liver parenchyma):

$$ \mathrm{CNR}=\left|\ {\upmu}_{\mathrm{R}}-{\upmu}_{\mathrm{L}}\right|/\mathrm{noise}\ \mathrm{liver} $$

### Statistics

All statistical analyses were performed using the software package JMP 11 (SAS Institute Inc., Vary, NC Arithmetic means (mean) and standard deviations (SD) were calculated and mean values were tested for statistical significant differences using a non-parametric Wilcoxon signed rank test. A *p*-value of less than 0.05 was considered significant.

Correlation analysis was performed using Spearman rank correlation coefficients. Spearman correlations are interpreted as follows in this study: |r| > 0.90 = very strong correlation; 0.6 < |r| < 0.9 = strong correlation; 0.4 < |r| < 0.6 = moderate correlation; |r| < 0.4 = weak correlation.

A Bland-Altman-analysis was performed for comparison of different measurement methods (arterial phase C-arm CT, PBV, CSI) of maximum tumor diameter. Mean difference (MD) and 95% confidence interval (CI) were determined.

## Results

### Subjective image quality analysis

According to a 5-point Likert scale, overall image quality of arterial phase C-arm CT acquisition and reconstructed PBV maps (*n* = 29) was good (4) or excellent (5) in 80% and in 97% of the cases, respectively. A significant better image quality of the PBV maps compared to arterial phase C-arm CT acquisition (*p* = 0.0032) was observed. None of the C-arm CT acquired arterial phase or PBV maps showed a poor (2) or non-diagnostic (1) image quality.

As displayed in Table 2, arterial phase acquisitions showed a significantly higher amount of noise and artifacts compared to PBV maps (*p* = 0.195 and *p* = 0.0001). None of the acquisitions and/or reconstructions showed major artifacts affecting diagnostic image quality. Central hepatic arteries showed significantly sharper delineation in PBV maps compared to arterial phase C-arm CT acquisitions (*p* = 0.0213), whereas the wall of the gallbladder tended to be more clearly delineated in arterial phase C-arm CT images, however this finding was not significant (*p* = 0.1353). Mean values, standard deviations and *p*-values of the investigated parameters are listed in Table 2.

**Table 2:** Image quality analysis using 5-point Likert Scale

Parameter	C-arm CT arterial phase	PBV map	*p*-value
Overall quality (*n* = 29)	3.93 (±0.59)	4.34 (±0.55)	0,0032*
Artifacts (*n* = 29)	2.79 (±0.56)	2.21 (±0.56)	0,0001*
Gallbladder wall (*n* = 27)	4 (±0.73)	3.7 (±0.61)	0,1353
Large vessels (*n* = 29)	4.14 (±0.52)	4.48 (±0.51)	0,0213*
Noise (*n* = 29)	3.48 (±0.57)	3.79 (±0.41)	0,195

* statistically significant

Complete acquisition of the liver was achieved in 59% (*n* = 17) of all cases. However, the diagnostic quality (1) was not restricted by missing parts of the liver in any of the affected cases due to exact patient positioning in knowledge of the localization of the lesions from the pre-interventional imaging. Missing parts of the liver consisted of partly not covered segments 2 and 3 (*n* = 5), segments 5 and 6 (*n* = 2), segment 2 (*n* = 1), segment 4 (*n* = 1), segment 6 (*n* = 1), segment 7 (*n* = 1), and segment 2, 3, 5, and 6 (*n* = 1).

### Lesion characterization and detection of tumor feeding vessels

A total of 52 hypervascularised HCC lesions were detected in arterial phase C-arm CT and reconstructed PBV maps. Mean diameter of HCC lesions was 26.6 mm (±15.8 mm) in arterial phase C-arm CT and 24.9 mm (±15.1) in PBV maps resulting in a significantly higher diameter in arterial phase C-arm CT images (*p* = 0,0004). An excellent correlation was seen between both methods (rho = 0.93). The lesions showed similar, but significantly higher conspicuity in PBV maps. Mean conspicuity was 4.27 (±0.74) in PBV maps as opposed to 3.83 (± 1.08) (*p* < 0.0001) in arterial phase images. Both methods showed a strong correlation (rho = 0.74). Calculated CNR was significantly higher in PBV maps compared to arterial phase C-arm CT with a mean CNR of 22.84 (±39.75) compared to 3.39 (± 2.51) (*p* < 0.0001). Regarding detectability of tumor feeders, significantly more feeding vessels were detected in arterial phase C-arm CT (1.67 ± 0.92) compared to PBV maps (1.27 ± 0.63) (*p* = 0.0001).

### Comparison to cross-sectional imaging

**Fig. 1:**
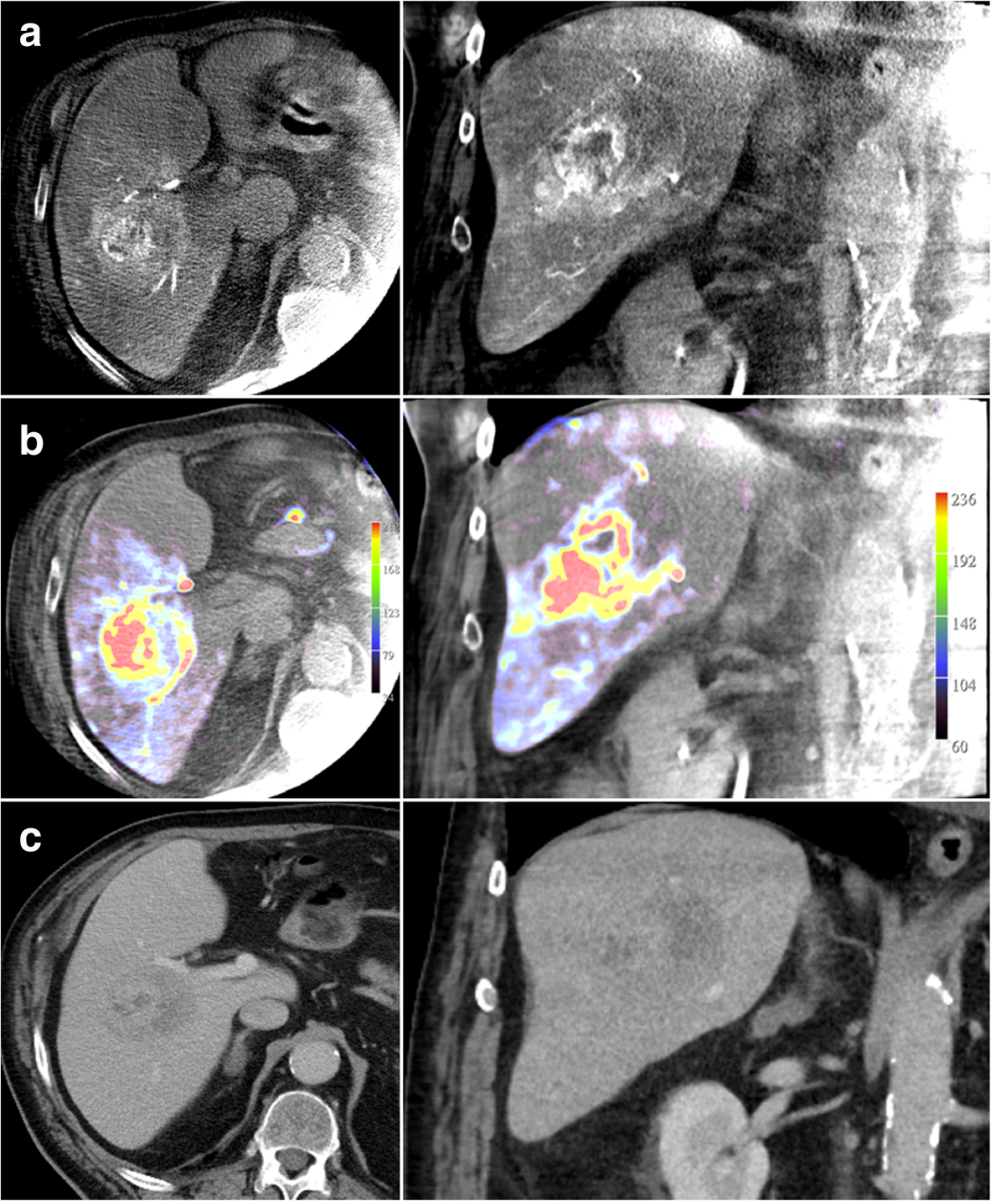
Images of HCC lesion at liver segment V of a 82-year old male patient in arterial phase (**a**), PBV map (**b**) as well as pre-interventional CT at portal venous phase (**c**)

Of 52 HCC lesions detected on CBCT imaging, a corresponding lesion in pre-interventional cross-sectional imaging was found in 51 cases (98%). One lesion was not seen in pre-interventional CT imaging. An example of arterial phase imaging, PBV map and CSI imaging is given at Fig. 1.

The maximum diameter of HCC lesions in pre-interventional CSI was 25.2 mm (±15.1) compared to 24.9 mm (±15.2) in PBV (*p* = 0.20, MD = − 0.25 mm, CI = − 0.73 to 0.22 mm). Maximum tumor diameter in arterial phase C-arm CT (26.5 ± 15.9) was significantly higher compared to CSI (*p* = 0.0212, MD = 1.27 mm CI = 0.11 to 2.44 mm).

**Fig. 2:**
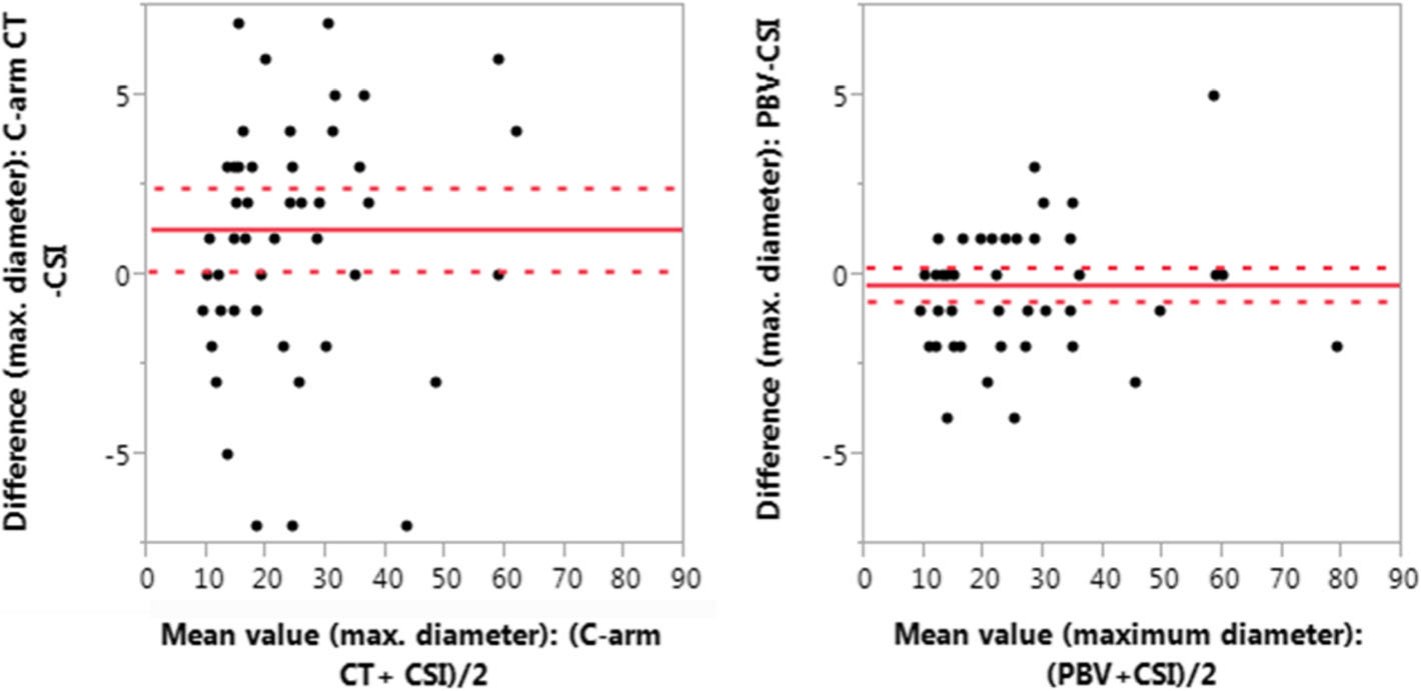
Bland-Altman plot providing maximum tumor diameter measured at C-arm CT and CSI

Results of the Bland-Altman plot are shown in Fig. 2. Spearman’s correlation coefficient rho revealed an excellent correlation between maximum tumor diameter in CSI and arterial phase C-arm CT (rho = 0.9168) as well as in CSI and PBV maps (rho = 0.9814).

## Discussion

Due to the opportunities of peri-interventional 3D imaging in combination with high contrast resolution and high spatial resolution of the arterial vasculature, the use of C-arm computed tomography during TACE treatments of HCC has steadily increased in recent years. Numerous studies have evaluated its clinical usefulness [9, 18] and hence the Cardiovascular and Interventional Radiological Society of Europe (CIRSE) as well as the Society of Interventional Radiology (SIR) recommend its use in TACE procedures [19].The exact localization of tumor is crucial to increase the selectivity of drug delivery into the targeted tumor tissue, thereby limiting non-target embolization and preserving healthy liver tissue. Eventually, such an approach will optimize tumor response to liver directed therapy [20]. However, detection of small or less vascularized tumors is reported to be limited using conventional angiography, especially in advanced cirrhosis with heterogeneous perfused liver parenchyma [21]. C-arm CT allows intra-procedural acquisition of arterial phase images in a volume of interest, whereas dual phase C-arm CT enables acquisition of the liver parenchyma during different parenchymal phases and the acquisition of parenchymal blood volume (PBV) information as well as perfusion data [11, 22].

As compared to conventional CT acquisition, C-arm CT is associated with limitations concerning image quality, mostly due to the increased photon scatter and increased image noise generated by the small acquired FOV [22]. This disadvantage might result in an incomplete visualization of the entire liver, as it was seen in 12 (41%) of our patients. However, due to correct patient positioning, all HCC lesions could be detected in this study. The assessment of overall image quality revealed a significantly superior image quality of PBV maps compared to arterial phase C-arm CT acquisition as well as lower amount of noise and artifacts, which is most likely related to advanced post-processing techniques involved in the generation of PBV maps compared to the arterial phase images.

The high spatial resolution of C-arm CT due to the flat detector technology in conjunction with improved depiction of soft tissue details has already demonstrated an improved visibility of small HCCs [23] and direct contrast agent administration to hepatic arteries results in markedly higher tumor-to-liver contrast ratio compared to CSI [22]. Loffroy et al. [7] investigated detectability of HCC lesions by C-arm dual-phase CT compared to contrast enhanced MRI and showed that dual-phase C-arm CT is more useful and reliable than single-phasic imaging to depict HCC lesions. Lucatelli et al. [8] found that C-arm CT has a significantly higher diagnostic performance detecting smaller or less vascularized lesions than multidetector CT. In contrast to this data, our retrospective study investigates the value of arterial phase images and PBV maps acquired with CBCT during TACE treatment as compared to pre-interventional cross-sectional imaging modalities.

In this study, we found a higher conspicuity of tumor lesions in PBV maps compared to arterial phase images, which is most likely explained by the above mentioned improved image quality of PBV maps compared to arterial phase images. Furthermore, lesion size was slightly overestimated in arterial phase images compared to PBV maps and CSI. This finding was in line of the study published by Tacher et al. [24] using a semiautomatic tumor segmentation software in measuring tumor volume on CE-MRI and dual phase CBCT images. For patient outcomes, the exact detection and treatment of tumor lesions and its feeders is of utmost importance [25]. Using C-arm CT during TACE, one lesion was detected (size 32x22mm) which had been missed by pre-interventional multiphasic CT. This is likely related to the higher tumor-to-liver contrast ratio using CBCT. This observation is concordant with the literature. Several studies showed an equal or even better sensitivity of tumor detection of HCC in C-arm CT compared to MDCT [26], especially in smaller tumors.

Virmani et al. investigated the utility of C-arm CT to optimize the catheter position during TACE and correction of catheter position was necessary in almost 39% based on C-arm CT assessment [27]. Traditionally, the detection of tumor feeders has been performed using conventional fluoroscopy and DSA. However, due to the two-dimensional character of this technique, the detection is limited by the potential misidentification or poor visualization of tumor feeders, mainly due to superimposed vessels. This could result in unnecessary or insufficient treatment, thereby potentially negatively impacting the individual patient outcome. Moreover, it has been demonstrated that in a significant percentage of cases, MDCT and MRI cannot correctly define the intra- and/or extrahepatic arterial feeders [28]. C-arm CT offers a 3-dimensional visualization of the liver vessel with high vessel-to-liver contrast resolution and high spatial resolution. Therefore, sensitivity for detecting tumor feeders has been found to range between 73 and 100% [6, 29]. These results are supported by this study which showed significantly more feeding vessels in the C-Arm CT arterial phase compared to PBV maps and CSI, probably due to the higher contrast concentration caused by direct intra-arterial injection of contrast material. This leads to markedly improved visualization of smaller vessels compared to CTA acquired in MDCT during intravenous application of the contrast agent. Furthermore, volume rendering and planar reformats that complement digital subtraction angiography allow for clarification of three-dimensional vascular relationships and provide a road map that simplifies the complex vascular anatomy in cirrhotic patients [30].

In summary, whereas overall image quality, conspicuity of HCC lesions and determination of tumor size was better in PBV maps, arterial phase images are necessary in order to accurately detect tumor feeders.

The study has several limitations. First, the study has a retrospective design and the size of the patient cohort is limited. Therefore, these results have to be interpreted with care and cannot be transferred to other C-arm CT vendors and protocols. Another possible limitation is the use of two different modalities in pre-interventional imaging, which is due the retrospective study design. However we only used 1.5 T scanners for MRI imaging and 128 slices scanners for CT. Subgroup analysis of the two different imaging techniques didn’t show any significant difference in lesion diameters between CT vs CBCT and MRI vs CBCT.

## Conclusion

The combination of PBV maps and arterial phase images acquired by C-arm CT during the TACE procedure enables for precise detection of the majority of HCC lesions and tumor feeding arteries and may therefore potentially increase patient outcomes.

## Abbreviations

BCLC: Barcelona Clinic Liver Cancer
CBCT: Cone beam computed tomography
CBCTHA: Dual-phase cone-beam computed tomography
CIRSE: Cardiovascular and Interventional Radiological Society of Europe
CNR: Contrast to noise ratio
CSI: Cross sectional imaging
CT: Computed tomography
CTA: Computed tomography angiography
DSA: Digital subtraction angiography
HCC: Hepatocellular carcinoma
HIPAA: Health Insurance Portability and Accountability Act
MDCT: Multidetector computed tomography
MRI: Magnetic resonance imaging
NASH: Nonalcoholic steatohepatitis
PBV: Parenchymal blood volume
SD: Standard deviation
TACE: Transarterial chemoembolization

## References

[1] Maillard E (2011). Epidemiology, natural history and pathogenesis of hepatocellular carcinoma. Cancer Radiother.

[2] Llovet JM, Bru C, Bruix J (1999). Prognosis of hepatocellular carcinoma: the BCLC staging classification. Semin Liver Dis.

[3] European Association For The Study Of The L, European Organisation For R, Treatment Of C: EASL-EORTC clinical practice guidelines: management of hepatocellular carcinoma. J Hepatol. 2012;56:908–43. 10.1016/j.jhep.2011.12.001 22424438

[4] Bouvier A, Ozenne V, Aube C, Boursier J, Vullierme MP, Thouveny F, Farges O, Vilgrain V (2011). Transarterial chemoembolisation: effect of selectivity on tolerance, tumour response and survival. Eur Radiol.

[5] van den Hoven AF, Prince JF, de Keizer B, Vonken EJ, Bruijnen RC, Verkooijen HM, Lam MG, van den Bosch MA (2016). Use of C-arm cone beam CT during hepatic Radioembolization: protocol optimization for extrahepatic shunting and parenchymal enhancement. Cardiovasc Intervent Radiol.

[6] Iwazawa J, Ohue S, Mitani T, Abe H, Hashimoto N, Hamuro M, Nakamura K (2009). Identifying feeding arteries during TACE of hepatic tumors: comparison of C-arm CT and digital subtraction angiography. AJR Am J Roentgenol.

[7] Loffroy R, Lin M, Rao P, Bhagat N, Noordhoek N, Radaelli A, Blijd J, Geschwind JF (2012). Comparing the detectability of hepatocellular carcinoma by C-arm dual-phase cone-beam computed tomography during hepatic arteriography with conventional contrast-enhanced magnetic resonance imaging. Cardiovasc Intervent Radiol.

[8] Lucatelli P, Argiro R, Ginanni Corradini S, Saba L, Cirelli C, Fanelli F, Ricci C, Levi Sandri GB, Catalano C, Bezzi M. Comparison of image quality and diagnostic performance of cone-beam CT during drug-eluting embolic Transarterial chemoembolization and multidetector CT in the detection of hepatocellular carcinoma. J Vasc Interv Radiol. 2017; 10.1016/j.jvir.2017.03.008 28495451

[9] Tacher V, Radaelli A, Lin M, Geschwind JF (2015). How I do it: cone-beam CT during transarterial chemoembolization for liver cancer. Radiology.

[10] Kakeda S, Korogi Y, Ohnari N, Moriya J, Oda N, Nishino K, Miyamoto W (2007). Usefulness of cone-beam volume CT with flat panel detectors in conjunction with catheter angiography for transcatheter arterial embolization. J Vasc Interv Radiol.

[11] Chu WF, Lin CJ, Chen WS, Hung SC, Chiu CF, Wu TH, Guo WY (2014). Radiation doses of cerebral blood volume measurements using C-arm CT: a phantom study. AJNR Am J Neuroradiol.

[12] Vogl TJ, Schaefer P, Lehnert T, Nour-Eldin NE, Ackermann H, Mbalisike E, Hammerstingl R, Eichler K, Zangos S, Naguib NN (2016). Intraprocedural blood volume measurement using C-arm CT as a predictor for treatment response of malignant liver tumours undergoing repetitive transarterial chemoembolization (TACE). Eur Radiol.

[13] Peynircioglu B, Hizal M, Cil B, Deuerling-Zheng Y, Von Roden M, Hazirolan T, Akata D, Ozmen M, Balkanci F (2015). Quantitative liver tumor blood volume measurements by a C-arm CT post-processing software before and after hepatic arterial embolization therapy: comparison with MDCT perfusion. Diagnostic and interventional radiology (Ankara, Turkey).

[14] Zhuang ZG, Zhang XB, Han JF, Beilner J, Deuerling-Zheng Y, Chi JC, Wang J, Qian LJ, Zhou Y, Xu JR (2014). Hepatic blood volume imaging with the use of flat-detector CT perfusion in the angiography suite: comparison with results of conventional multislice CT perfusion. J Vasc Interv Radiol.

[15] Basile A, Carrafiello G, Ierardi AM, Tsetis D, Brountzos E (2012). Quality-improvement guidelines for hepatic transarterial chemoembolization. Cardiovasc Intervent Radiol.

[16] Sommer CM, Stampfl U, Kauczor HU, Pereira PL (2014). National S3 guidelines on hepatocellular carcinoma. Radiologe.

[17] Witjes CD, Willemssen FE, Verheij J, van der Veer SJ, Hansen BE, Verhoef C, de Man RA, Ijzermans JN (2012). Histological differentiation grade and microvascular invasion of hepatocellular carcinoma predicted by dynamic contrast-enhanced MRI. J Magn Reson Imaging.

[18] Pellerin O, Lin M, Bhagat N, Shao W, Geschwind JF (2013). Can C-arm cone-beam CT detect a micro-embolic effect after TheraSphere radioembolization of neuroendocrine and carcinoid liver metastasis?. Cancer Biother Radiopharm.

[19] Wallace MJ, Kuo MD, Glaiberman C, Binkert CA, Orth RC, Soulez G (2009). Three-dimensional C-arm cone-beam CT: applications in the interventional suite. J Vasc Interv Radiol.

[20] Mahnken AH, Spreafico C, Maleux G, Helmberger T, Jakobs TF (2013). Standards of practice in transarterial radioembolization. Cardiovasc Intervent Radiol.

[21] Sumida M, Ohto M, Ebara M, Kimura K, Okuda K, Hirooka N (1986). Accuracy of angiography in the diagnosis of small hepatocellular carcinoma. AJR Am J Roentgenol.

[22] Bapst B, Lagadec M, Breguet R, Vilgrain V, Ronot M (2016). Cone beam computed tomography (CBCT) in the field of interventional oncology of the liver. Cardiovasc Intervent Radiol.

[23] Tognolini A, Louie JD, Hwang GL, Hofmann LV, Sze DY, Kothary N (2010). Utility of C-arm CT in patients with hepatocellular carcinoma undergoing transhepatic arterial chemoembolization. J Vasc Interv Radiol.

[24] Tacher V, Lin M, Chao M, Gjesteby L, Bhagat N, Mahammedi A, Ardon R, Mory B, Geschwind JF (2013). Semiautomatic volumetric tumor segmentation for hepatocellular carcinoma: comparison between C-arm cone beam computed tomography and MRI. Acad Radiol.

[25] Lencioni R (2012). Chemoembolization in patients with hepatocellular carcinoma. Liver cancer.

[26] Zheng J, Li J, Cui X, Ye H, Ye L (2013). Comparison of diagnostic sensitivity of C-arm CT, DSA and CT in detecting small HCC. Hepato-Gastroenterology.

[27] Virmani S, Ryu RK, Sato KT, Lewandowski RJ, Kulik L, Mulcahy MF, Larson AC, Salem R, Omary RA (2007). Effect of C-arm angiographic CT on transcatheter arterial chemoembolization of liver tumors. J Vasc Interv Radiol.

[28] Matoba M, Tonami H, Kuginuki M, Yokota H, Takashima S, Yamamoto I (2003). Comparison of high-resolution contrast-enhanced 3D MRA with digital subtraction angiography in the evaluation of hepatic arterial anatomy. Clin Radiol.

[29] Minami Y, Yagyu Y, Murakami T, Kudo M (2014). Tracking navigation imaging of Transcatheter arterial chemoembolization for hepatocellular carcinoma using three-dimensional cone-beam CT angiography. Liver cancer.

[30] Pung L, Ahmad M, Mueller K, Rosenberg J, Stave C, Hwang GL, Shah R, Kothary N (2017). The role of cone-beam CT in Transcatheter arterial chemoembolization for hepatocellular carcinoma: a systematic review and meta-analysis. J Vasc Interv Radiol.

